# Community college student perceptions of digital anatomy models as a curricular resource

**DOI:** 10.1002/ase.2523

**Published:** 2024-10-15

**Authors:** Yvonne M. Baptiste, Samuel Abramovich

**Affiliations:** ^1^ Division of Business and STEM SUNY Niagara Sanborn New York USA; ^2^ Department of Learning and Instruction State University of New York at Buffalo Buffalo New York USA; ^3^ Department of Information Science State University of New York at Buffalo Buffalo New York USA

**Keywords:** 3D digital anatomy, curriculum, health professions education, instruction, student perceptions, teaching and learning technology, technology enhanced learning, technology in education

## Abstract

Digital model platforms and applications are common in anatomy education and continue to grow in number, which suggests that educators and students find use for these tools despite the lack of widely accepted best practices. Consequently, it is a challenge for educators to mindfully integrate digital models into curriculum. This short‐term, longitudinal study investigated the effects of integrating a monoscopic digital model as a teaching tool during lectures on reproductive and endocrine anatomy as an intervention in a community college human anatomy and physiology course. Student use and perceptions of digital models were analyzed for correlation with the nature of the course content and the intervention (*n* = 92). Academic content significantly affected self‐reported student use (*p* < 0.001) as well as student perceived usefulness of the model (*p* = 0.02). These findings support the conjecture that digital anatomy models may be better for achieving certain specific learning goals opposed to all learning goals. Integration of digital models as an instructional method did not consistently influence student behavior but it made a difference in participant ability to recognize this technology outside of the lecture. Overall, participants had a positive perception of digital models, although they were not perceived as more important than all other curricular resources. Inclusion of monoscopic digital models for teaching anatomy should be considered by educators since teaching with digital models can demonstrate strengths and weaknesses for students within the context the of learning objectives, assisting students to make more informed decisions about effective learning tools.

## INTRODUCTION

One objective of anatomy education is for students to acquire foundational knowledge about the structure of the human body. Anatomical knowledge is the underpinning of understanding normal physiologic processes and dysfunction.^1^, ^2^, ^3^, ^4^, ^5^ Ultimately for many students, anatomical knowledge enables safe clinical practices^6^ and is instrumental to students entering a healthcare profession.^7^ To acquire this knowledge, students use digital (e.g., virtual reality) and traditional (e.g., physical textbook, flashcards; cadaveric specimens) learning methods.^8^, ^9^, ^10^

Anatomy models are physical or digital entities that can enhance conceptual understanding and lead to knowledge construction.^11^, ^12^ Anatomy models have historically played a vital role for both learning and teaching in anatomy education^13^ because they are effective tools.^14^ In particular, both traditional and digital anatomy models are helpful for learning because they visually represent structures and relationships that are not readily or easily visible otherwise.^15^ Models also assist educators to teach anatomic concepts that are difficult to describe.^16^, ^17^, ^18^ The visual representation provided by models is indispensable for facilitating student comprehension.

The digitization of anatomy models has given rise to a vast array of digital curricular resources.^19^, ^20^ In brief, digital anatomy models are three‐dimensional (3D) interactive graphics that are bound to a screen or use virtual reality (VR)‐based technology. Digital models noticeably contained on a screen have been referred to as 3D visual technology (3DVT)^21^ and include mobile platforms (e.g., Primal Pictures, Informa UK Limited, London; Visible Body, MA; Zygote Body, Zygote Media Group, UT) and larger, more stationary systems (e.g., Anatomage, Anatomage, Inc., CA). Although 3DVT graphics give the appearance of 3D, screen‐bound anatomy models are considered monoscopic models because of the two‐dimensional (2D) nature of the screen.^22^ Virtual reality platforms offer anatomy models that appear to occupy 3D space in a realistic way (e.g., 3D Organon VR Anatomy, 3D Organon, CA; Human Anatomy VR, Virtual Medicine s.r.o., European Union). Virtual reality models are considered stereoscopic models due to the illusion that the model occupies 3D space rather than appearing to be screen bound. Research findings have shown that both monoscopic and stereoscopic digital anatomy models are effective learning tools.^23^, ^24^, ^25^

The overall purpose of using technology to transform traditional physical anatomy models and specimens into digitized versions is to evolve and improve the tool.^26^, ^27^ Digital anatomy models have some advantages over physical models and specimens, especially increased accessibility due to availability outside of the laboratory. This characteristic played a role in sustaining anatomy education during the Covid‐19 pandemic.^28^, ^29^ Another advantage of digital models is that they can be repeatedly deconstructed and reconstructed (i.e., virtual dissection) in a non‐destructive way, unlike cadaveric specimens. This creates an environment conducive to learning because mistakes are largely inconsequential since starting conditions can be reset. Digital models are more sustainable over a longer time period compared to cadaveric specimens. Digital models have played a significant role in transforming the landscape of anatomy education and will likely continue to do so.^30^, ^31^ Yet, despite these strengths, it remains debatable whether or not digital anatomy models are superior learning tools compared to their physical counterparts.^32^ For example, digital models may disadvantage learners with low visuospatial ability.^22^, ^33^ Additionally, digital models do not humanize anatomy education in the way that cadaver dissection does,^34^ which could result in less understanding about teamwork, responsibility, and compassion.^35^ Current research suggests that rather than digital models replacing physical models and specimens, these resources should coexist within an anatomy education curriculum because both are effective and relevant for different learning goals.^36^

### Digital anatomy models and pedagogy

If digital models should be a permanent addition to anatomy courses, then it is critical to understand how they can be purposefully integrated into anatomy education.^37^ Digital model platforms and applications continue to grow in number, which suggests that educators and students are finding use for these tools despite the lack of clearly defined best practices.^19^ However, the rapid proliferation of different types of digital anatomy models also makes it difficult for educators to keep up with the types, uses, advantages, and disadvantages of newer digital resources.^38^ This problem of knowing how and when to use digital models also exists for students. Learning human anatomy is an ambitious pursuit that most likely does not leave students extra time to figure out if and which digital model might best support various different learning goals. Educators commonly assist students with this dilemma by recommending specific curricular resources for achieving particular learning goals.^39^

One optimal way for educators to recommend a digital anatomy model is to incorporate it into the curriculum as a teaching tool during lecture and laboratory presentations. Three‐dimensional visual technology models enable an educator to present additional viewpoints that are not available in two‐dimensional curricular resources. Even with this potential advantage, mindfully integrating new teaching resources into a course curriculum is challenging for educators^38^, ^40^ for a number of reasons, including lack of training.^31^ Guidance to students from educators is necessary for the effective use of digital resources.^41^ For students, a common obstacle to using a 3DVT model is learning to navigate the digital interface.^42^, ^43^ Use of a 3DVT model for teaching has the potential to familiarize students with the resource.^44^ Student exposure to a 3DVT model during a lecture presentation could highlight advantages and disadvantages, thereby informing students whether or not 3DVT would assist their own study practices.

Although the efficacy of learning anatomy through student use of 3DVT anatomy models may be associated with personal characteristics of the learner such as spatial ability^22^ and visualization ability,^32^ letting students decide whether or not to utilize 3DVT as a study tool could help alleviate any unequal learning benefits among students. The success or failure of technology within a social setting like education will in part be determined by the social interactions, decisions, and perceptions of all stakeholders (e.g., students, educators, and administrators) rather than being strictly determined by the technological specifications.^45^, ^46^ Consequently, instructor demonstration of the model can establish the norms for these types of interactions by showing students what is possible with 3DVT models. In this study, a monoscopic, 3DVT anatomy model was utilized as a teaching tool during lecture presentations as a light touch intervention.

Educational interventions frequently target student thinking, particularly in areas such as improving student self‐efficacy and encouraging positive attitudes toward learning.^47^, ^48^ Light touch interventions such as positive affirmations have been used successfully in undergraduate education to improve grades.^49^, ^50^ By teaching with a 3DVT model as an academic intervention, it repeatedly demonstrated the model to students in a purposeful way.^51^ The intent of this pedagogy was to help students understand how, why, and when 3DVT might assist learning. The intervention was also designed to address the possibility that students would not use the 3DVT anatomy model as a study tool if they did not understand the potential value of the resource. In other words, the intervention was designed to increase familiarity (i.e., assist students to become more knowledgeable about how to access and use the 3DVT model). Familiarity has been shown to be an important characteristic of student‐chosen resources.^44^

Previous research studies have resulted in contradictory findings regarding the effectiveness of 3DVT for learning anatomy.^25^ Student use of 3DVT has demonstrated improved learning in some cases,^52^ yet offered no advantage in other cases.^33^ Since there is no clear consensus about best practices for 3DVT use in anatomy education, using it as a teaching tool may help to inform both the educator and student about affordances and constraints of 3DVT anatomy models for any given academic content. If students had increased familiarity with the 3DVT anatomy model, they could better determine which study resources would be most effective to achieve their learning goals, whether that included the use of 3DVT or not.^53^, ^54^

The primary objective of this study was to investigate whether or not incorporating a 3DVT anatomy model for teaching purposes influences student use or perception of 3DVT anatomy models in a community college human anatomy and physiology (HAP) lecture course. It was hypothesized that if students were familiarized with 3DVT models during lecture presentations, then they would access 3DVT models more. Three research questions guided the experimental design, data acquisition, and analysis:

1. Is student use of a 3DVT model associated with teaching methodology and/or academic content?
2. What is student perception of the 3DVT anatomy model as a study tool and how does it compare to other curricular resources?
3. Are there measurable student benefits when a 3DVT model is integrated into the curriculum as a teaching tool as evidenced by improved academic performance?

## METHODS

This study used a nonrandomized interventional design and took place within an educational setting (i.e., participants were actively enrolled in the course for a grade). This design was advantageous because it offered contextual relevance to the data.^55^ Participants in this study, just like any student, had the intent to successfully complete the course.^56^

### Human anatomy and physiology course design

The course selected for this study was human anatomy and physiology II (HAP2) lecture, the second half of a two‐semester sequence (preceded by human anatomy and physiology I lecture) offered at a community college. The course was required for various academic programs such as registered nursing and allied health fields (e.g., radiologic technologist, surgical technologist, and medical assistant). The course was 15 weeks long with four lecture hours per week, totaling 60 h of instruction per course. Academic content in HAP2 was organized by body system and included the reproductive (12 h), endocrine (10 h), cardiovascular (12 h), respiratory (8 h), urinary (10 h), and digestive systems (8 h).

This study took place during the first one third of the HAP2 course, consisting of 5.5 weeks (22 h) of instruction. Two academic content units were included in this study, reproductive and endocrine systems. The study was planned to run the entire semester, covering all academic units, but the Covid‐19 pandemic altered this plan. Reproductive system topics taught using 3DVT during this study included gonadal anatomy along with internal and external accessory structures and mammary gland anatomy. Endocrine gland anatomy was also taught using 3DVT models of major endocrine structures such as the hypothalamus, pituitary gland, pineal gland, thyroid gland, thymus, pancreas, and adrenal glands.

Curricular resources other than the 3DVT anatomy model were also part of the course. These resources included a required textbook, Human Anatomy and Physiology, 11th Ed.^57^ Lecture notes outlining the topics covered in class were also provided by the instructor and cross‐referenced to the relevant textbook sections. A digital study area accompanied the textbook and was available to all students. It included video, simulation, flashcards, animation, art‐labeling activities, and practice tests and quizzes. One required homework per academic unit was assigned using a digital platform, Mastering A&P (Pearson Higher Education). Use of all resources was optional, except for the textbook and one required homework assignment per academic unit.

Both HAP2 and its preceding lecture course had a co‐requisite laboratory component. Different laboratory sections were taught by different instructors but all laboratory sections used the same syllabus, learning objectives, and methodologies. Each participant was enrolled randomly in the co‐requisite laboratory portion of this course. However, only the lecture section of the course was included in this study because lecture was the modality used to teach anatomic concepts whereas the laboratory was primarily used for hands‐on activities that reinforced academic content covered in the lecture. Examples of hands‐on laboratory activities included examination of microscope slides and physical models, wet laboratory investigations (e.g., blood typing and urinalysis) and organ dissections (e.g., sheep heart and sheep kidney).

### Participants

Convenience sampling was utilized since it would generate the necessary participation for analysis without negative impacts such as bias or low power analysis. All students who were enrolled in one of four traditional, face to face sections of a HAP2 lecture course were invited to participate in this study. All participants were assumed to be in either the first or second year of college since the course was held at a 2‐year institution.

The criteria for participation were completion of the prerequisite course and being at least 18 years of age. The participants were recruited through written information distributed in the classroom and a post within the course management system (CMS), that is, Blackboard Learn (version 9.1). An Institutional Review Board (IRB) determined this study to be exempt. Written informed consent was obtained from all participants.

All surveys were administered using Qualtrics XM (Qualtrics, Provo, UT) during January–March 2020. Students were awarded 2 points on the unit test grade as compensation for completing a survey. Congruous with ethical conduct, all students were eligible to complete the surveys and earn the bonus points, but only survey responses from participants who signed informed consent to participate in this study were analyzed. Grades analyzed in this study did not include the bonus points awarded for survey completion.

### Study design and group allocation

Participants were divided into two groups: Control and intervention. Participant allocation was non‐randomized. Participants were assigned to groups based on the lecture section they were enrolled in. The four lecture sections included in this study were academically similar. The groups each had the same syllabus, course objectives, expected student learning outcomes, assessments, and instructor which lessened variability as a source of error.

### Educational intervention

The only difference between the control and intervention groups was that the intervention group lecture presentations included use of a 3DVT model projected onto a large screen. It was used as a visual tool during lecture to illustrate relevant anatomy being covered in class, in addition to traditional teaching tools such as 2D graphics. Unlike static 2D images that are traditionally used in anatomy education, the 3DVT anatomy model was dynamic. The 3DVT model was used by the educator during intervention group lecture presentations to reveal complex anatomical relationships from various angles and depth levels. Different types of interactions such as zoom and ghosting (i.e., solid structure made transparent) were used to illustrate anatomical relationships that are hard to see otherwise. For example, the spatial relationships among the bladder, urethra, and prostate gland were demonstrated during lecture using the 3DVT model.

**FIGURE 1:**
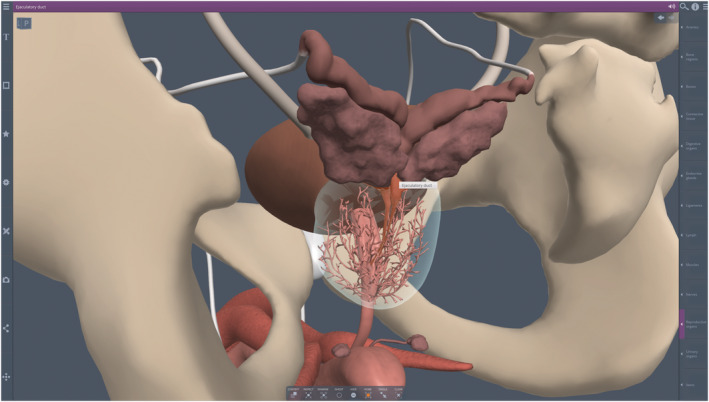
Screenshot of the digital anatomy model user interface. Representative posterolateral view of male pelvis with selected anatomy including skeletal, reproductive, and urinary components. The prostate gland is ghosted to show the contained duct system, ejaculatory ducts (highlighted and labeled), and urethra.

The 3DVT anatomy model utilized in this study was Anatomy.tv, a licensed resource offered by the college library, accessible to all students and faculty (3D Real‐time Human Anatomy. © Pharma Intelligence UK trading as Primal Pictures, a Citeline business, 2024) (Figure 1). The same 3DVT model used in class for teaching the intervention group was available to both control and intervention group members whether they chose to participate in the study or not, although student use of 3DVT was not required by either participant group.

The 3DVT model was interactive, and had features such as free rotation, zoom, pan, labels, pronunciations, ghosting, and deleting among other capabilities. The 3DVT model could be accessed on a wide variety of electronic devices. To make the 3DVT anatomy model easy for all students to access, a link was available in the CMS. The 3DVT model was also accessible to students through the college library website directly. The model was fully functional both on and off campus, so it could be utilized at a time and place convenient to the student.

### Data sources

**FIGURE 2:**
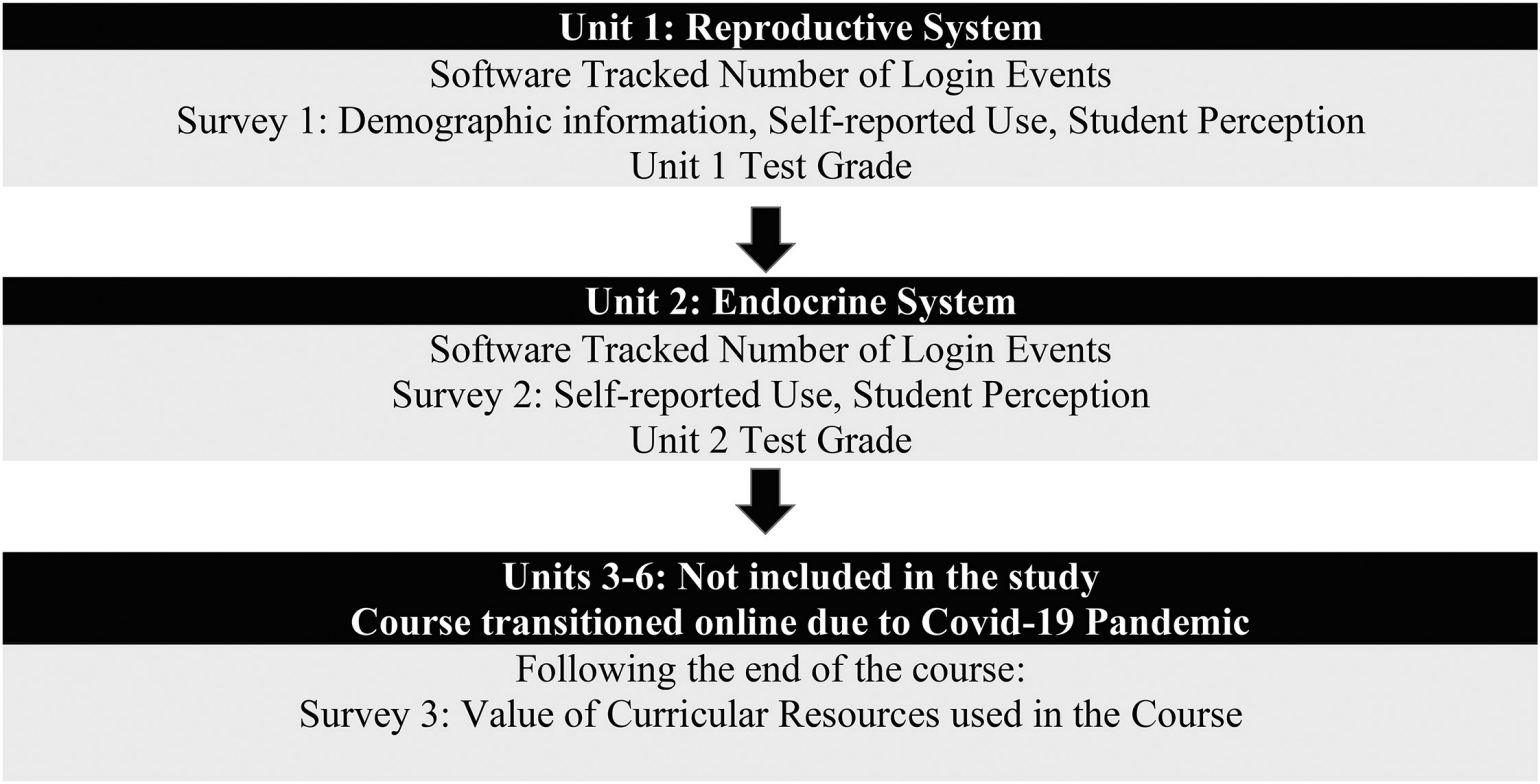
Research timeline: Summary of the data types and timing of data collection across the study.

To address the research questions, this study collected multiple different types of data, including the number of 3DVT login events, participant self‐reported voluntary use of 3DVT, participant perception of 3DVT value as study tool, and unit test grades. All data sources utilized in this study were easily accessible within the community college environment. Figure 2 outlines the timing and types of data collection that occurred throughout the study.

#### Use data

Participant access and use of 3DVT as an optional study tool was measured in two ways: tracking login events and participant self‐reports. Both these data sources were used to address research question 1: Is student use of a 3DVT model associated with teaching methodology and/or academic content? Two data sources were used to limit bias inherent in a single unit of measure. Even though both data sources measured 3DVT use by participants, they measured different aspects of use. The login event report corresponded to the behavior of accessing 3DVT, while self‐reported measures were participant generated estimates of the number of times the participant used the 3DVT model per academic unit that likely correlated with student perceived alignment with learning outcomes.^58^ Self‐reported data have been shown to be accurate measures for research purposes.^59^, ^60^, ^61^

##### Login events to the 3DVT model

Each time a participant clicked the CMS link to the 3DVT model it counted as a login event. A report was generated showing number of login events per day per participant for both academic content units included in this study. Although this data did not include login events outside the CMS (i.e., direct access through the library website) or connection time, it provided a consistent and reliable way to track participant behavior.

##### Participant self‐reported use of 3DVT

Self‐reported use was collected using survey items at the conclusion of each academic unit. Further description of these items can be found in section: Surveys 1 and 2.

#### Perception data

Student perception of the 3DVT model was collected using survey items. Student perceived value of the 3DVT model specific to a particular academic unit was collected at the conclusion of each academic unit using an ordinal scale survey item.

Student perception about strengths and weaknesses of the 3DVT model, overall importance of the 3DVT model, and how the 3DVT anatomy model compared to other curricular resources available was collected at the end of the course using both ordinal scale and open‐ended survey items described further in section: Survey 3. Student perception data was used to address research question 2: What is student perception of the 3DVT anatomy model as a study tool and how does it compare to other curricular resources?

#### Unit test grades

Unit test grades were used to address research question 3: Are there measurable benefits when a 3DVT model is integrated into the curriculum as a teaching tool as evidenced by improved academic performance? The unit tests that were administered to both the control and intervention groups were identical and consisted of questions answered using a scannable answer sheet.

### Survey composition

In total, three surveys designed by the researchers were used in this study. Surveys 1 and 2 were short surveys administered following each academic unit and focused on collecting data pertaining to that particular academic unit. Survey 3 was an exit survey administered at the conclusion of the semester. It collected participants' overall opinion about 3DVT anatomy models and how they compared to other available study tools. All surveys were designed to be short reports of 3DVT use and usefulness. While not traditionally validated, non‐validated measures can be acceptable instruments^9^, ^62^ when used in education research. Although validated questionnaires measuring student perceptions within specific educational settings are becoming more available,^63^ validated instruments are not always available for specific contexts and qualitative exploration. To address potential bias introduced by using non‐validated surveys, a description of each survey is provided.

#### Surveys 1 and 2

Surveys 1 and 2 contained the same items, with the exception of demographic items that were also included in Survey 1. The demographic questions contained in Survey 1 were adapted from the Community College Survey of Student Engagement, a validated survey developed by the National Survey of Student Engagement. The demographic information requested of participants included age, academic credentials, nonacademic responsibilities (e.g., work and dependents), and academic goals.

Other than the demographic questions, Surveys 1 and 2 consisted of the same four questions. Two items asked the participant to report how much they used the 3DVT model to study for that particular academic unit. The self‐reported use item analyzed in this study had a 5‐point ordinal response scale^64^ ranging from no use to heavy use (over 10 uses).

Student perception about the value of the 3DVT model was also collected by Surveys 1 and 2. Participants were asked to grade the 3DVT model as an optional study resource for that particular unit of study. A 4.0 grade point average (GPA) scale was utilized as the response scale. It was a parametric scale that ranged from A to F, with A being the highest grade (4.0 value) and F being the lowest grade (no value). The advantages of this measure included the timing of data acquisition (i.e., data were collected immediately at the conclusion of the individual academic unit) and the intuitive response scale. The response scale of A–F was familiar to students because it was the same scale used by the institution to grade courses and consisted of a robust 12‐point scale. An increased number of response categories has been shown to improve the measurement,^65^ as long as the number of choices is not overwhelming to the participant.

Participants were also surveyed to confirm that both control and intervention group members had equivalent exposure to 3DVT in the laboratory. The item asked whether or not the 3DVT model was used by the instructor in the laboratory section. The response choices were nominal: Yes, No, and Unsure. The purpose of this survey item was to check any potential bias regarding the control and intervention conditions.

#### Survey 3

The purpose of Survey 3 was to gain an understanding of participant perceptions regarding strengths and weaknesses of 3DVT, and to compare 3DVT to other available curricular resources used in the course. In total, Survey 3 consisted of 10 ordinal response items and 2 open‐ended questions.

To gauge student perception about specific strengths and weaknesses of the 3DVT model, an open‐ended question asked, “Which parts of the 3D anatomy model were useful or beneficial to you? Which parts were not useful or problematic?”

To contextualize student perceptions about the 3DVT anatomy model, participants compared the 3DVT model to other curricular resources available in the course. This was done by asking participants to rate the level importance of five curricular resources available within the course: (1) the 3DVT model, (2) the textbook, (3) lecture notes, (4) digital study area, and (5) homework assignments. All responses were collected on the same 5‐point ordinal scale that ranged from not at all important to extremely important. Ratings for the five resources were collected only for each academic unit included in this study (i.e., reproductive and endocrine systems). Additionally, an open‐ended question was posed that did not mention the 3DVT model. Specifically, “Overall, how important was the use of supplemental material in this course?”

## DATA ANALYSIS

Both quantitative and qualitative methods were utilized for data analysis. Quantitative methods were used to examine student logins to the 3DVT model, unit test grades, and scaled survey responses. Qualitative methods were used to examine open‐ended survey responses. Given that participation rates typically decrease over the course of a semester,^66^ some level of survey attrition was assumed. Therefore, statistical methods resilient to varying participation rates were selected.

### Quantitative methods

Parametric data (i.e., the number of login events, the student assigned grade reporting 3DVT value, and unit test grades) were used to make comparisons between control and intervention groups using independent *t*‐tests. Between‐group analyses were evaluated using Levene's test of equality of variances prior to analysis. Related‐samples analyses excluded pairs with missing data points.

Since unit test grades were used to analyze if measurable differences in academic performance could be detected between the control and intervention groups, baseline academic equivalence between the control and intervention group participants was examined by comparing the final grades earned in the prerequisite course, human anatomy and physiology 1 lecture. This analysis was done by transforming the participants final letter grade in the prerequisite course to grade points and performing an independent *t*‐test comparing control and intervention groups. This initial analysis was done to provide context to other analyses regarding potential grade differences between control and intervention group participants.

Nonparametric data (i.e., ordinal responses) used to make comparisons between control and intervention group participants were analyzed using the Mann–Whitney test. Repeated measures were analyzed using Wilcoxon signed‐rank test. Nominal survey responses were first checked for tenable assumptions (i.e., percentage of cells have expected count less than 5) and then analyzed using Pearson's chi‐square test.^67^

Quantitative analyses were performed using IBM SPSS Statistics for Macintosh, version 29 (IBM Corp., Armonk, NY). Alpha level 0.05 was used for analyses. A Bonferroni correction was applied when analyzing repeated measures, requiring a stricter *p* value for significance.

### Qualitative methods

Qualitative methods were used to collect, analyze, and interpret the open responses from Survey 3. A deductive content analysis was used to first organize the data and then code the comments.^68^ Data were initially assigned to five codes based on the research questions, and then subcategorized based on themes and contrasts.^69^ Since the amount of qualitative data collected in this study was relatively small, the coding was done by a single researcher. This reduced threats to internal validity and eliminated the need for evaluating inter‐rater reliability. To address and limit potential bias inherent with a single coder, all codes including code definitions and an example verbatim citation for each code were examined by three reviewers. ATLAS.ti Scientific Software Development GmbH (Version 9.1.3 Mac) (Berlin, Germany) was used to track, code, help uncover and analyze the complex phenomena contained in the unstructured data. All qualitative data entry and analyses were performed following the conclusion of the course in an effort to limit bias.

## RESULTS

### Descriptive data

In total, 112 students were enrolled across all four lecture sections included in this study. Ninety‐two students participated (82% participation rate). The control and intervention groups consisted of two traditional, in‐person lecture sections. The control group had a total of 46 participants (84% participation rate) and the intervention group also had a total of 46 participants (81% participation rate). Participants are described in Table 1.

**TABLE 1:** Participants' demographic information.

Characteristics	Participants *n* (%)	
	Control	Intervention
Sex		
Female	39 (84.8%)	33 (71.7%)
Male	7 (15.2%)	13 (28.3%)
Age in years; mean (±*SD*)	25.7 (±7.4)	25.3 (±7.5)
Dependent children	11 (23.9%)	10 (21.7%)
Work for pay	42 (91.3%)	42 (91.3%)
Academic program		
Liberal Arts and Sciences	23 (50%)	29 (63%)
Surgical Technologist	6 (13%)	8 (17.4%)
Registered Nursing	6 (13%)	3 (6.5%)
Other	3 (6.5%)	5 (10.9%)
Physical Therapists Assistant	4 (8.7%)	1 (2.2%)
Medical Assistant	2 (4.3%)	0
Licensed Practical Nurse	1 (2.2%)	0
Massage Therapist	1 (2.2%)	0

*Note*: Summary of participant (*n* = 92) responses, (±*SD*) = ± standard deviation.

Participant response rates declined over the course of the study. Survey 1 was administered at the start of the semester and had a 100% response rate (*n* = 92), although 7.6% of Survey 1 responses were incomplete. The response rate for Survey 2 was 83.7% (*n* = 77), 100% of responses were complete. Survey 3 was administered at the end of the semester and had the lowest response rate, 80.4% (*n* = 74), 100% complete. Participant abstention from submitting a survey and incomplete survey submissions were reported as missing data.

### Student logins to 3DVT anatomy model

Analysis of the number of logins events tracked by the link provided in the CMS revealed no significant difference in login activity.

A comparison of the number of login events to the 3DVT model over the course of the study indicated no significant difference between control and intervention participants. Levene's test confirmed variances between groups was not significantly different (*p* = 0.8), indicating the assumption of homogeneity was met. The control group participants (*M* = 24.76, *SD* = 22.69) and the intervention group participants (*M* = 25.97, *SD* = 19.63) logged into the 3DVT model using the link provided in the CMS a similar number of times, (*t*(90) = −0.275, *p* = 0.78) across the length of the study.

A related‐samples comparison of the total number of login events for all participants (control and intervention groups combined) between academic units showed that number of logins occurring during the reproductive system unit (*M* = 12.6, *SD* = 12) and endocrine system unit (*M* = 12.8, *SD* = 11.5) was not significantly different (*t*(91) = −0.24, *p* = 0.81).

Additionally, between groups analyses per academic unit were also evaluated. Levene's test confirmed the assumption of homogeneity was met for both unit 1 (*p* = 0.65) and unit 2 comparisons (*p* = 0.79). During unit 1, the reproductive system, the control group participants logged into the 3DVT model (*M* = 12.6, *SD* = 13.35) a similar number of times to the intervention group participants (*M* = 12.5. *SD* = 10.72, *t*(90) = 0.043, *p* = 0.966). A similar lack of difference in the number of logins was observed during unit 2, the endocrine system. The control group participants (*M* = 12.2, *SD* = 11.35) and intervention group participants (*M* = 13.47, *SD* = 11.65) logged into the 3DVT model about the same number of times (*t*(90) = −0.553, *p* = 0.58).

### Self‐reported student use of 3DVT anatomy model

**FIGURE 3:**
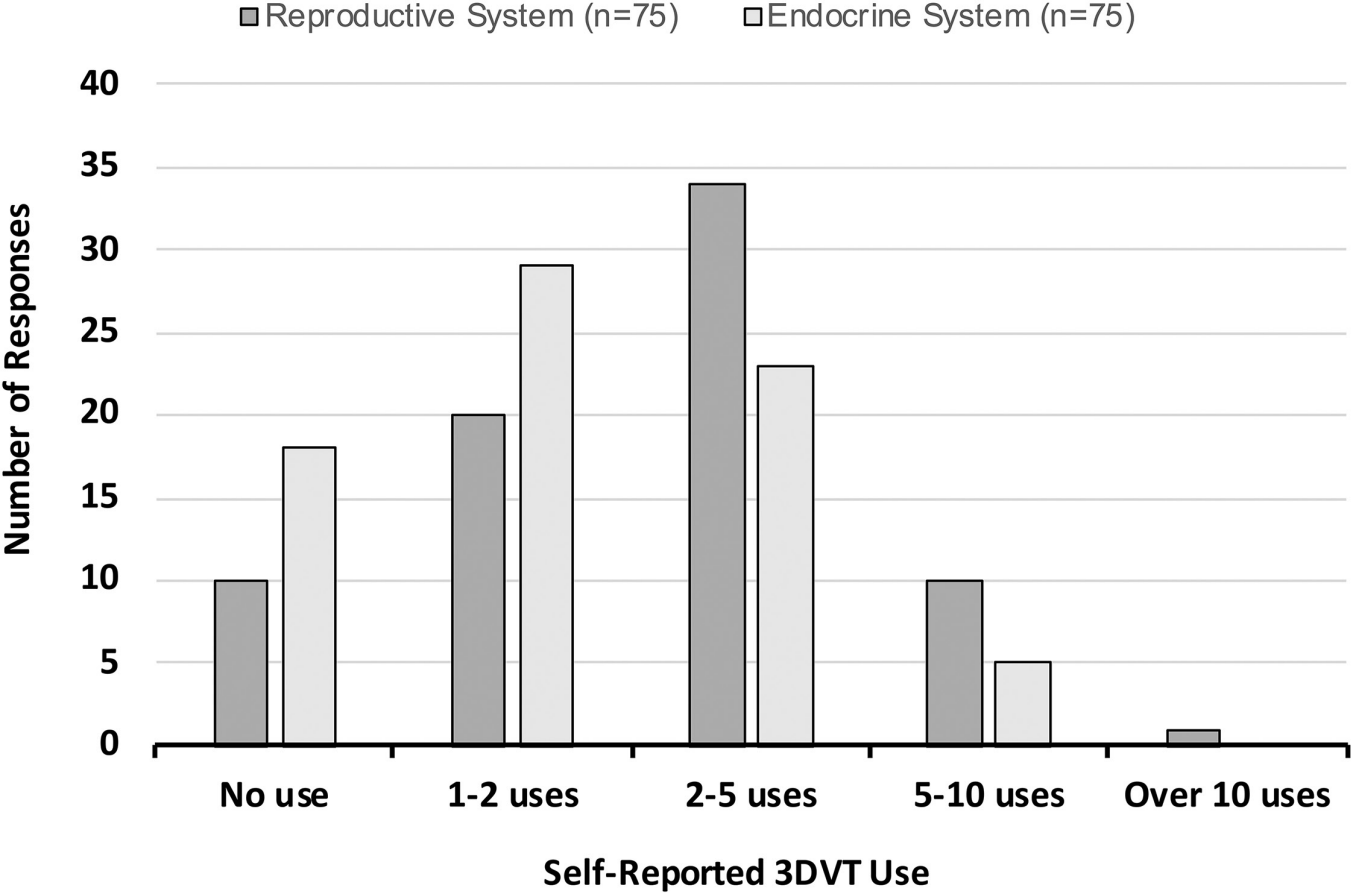
Comparison of self‐reported 3DVT use per academic unit. Frequency distribution of participant self‐reported 3DVT use for the reproductive system (*n* = 75) and the endocrine system units (*n* = 75).

A related‐samples Wilcoxon signed‐rank test revealed that the academic unit was a significant factor for participant reported 3DVT model use. Participants (*n* = 75, Excluded incomplete cases = 17) reported using the 3DVT model as a voluntary study tool significantly more for the reproductive system (*Mdn* = 2) than for the endocrine system (*Mdn* = 1; *T* = 118.5, *p* < 0.001) with a medium effect size (*r* = −0.47) (Figure 3).

**FIGURE 4:**
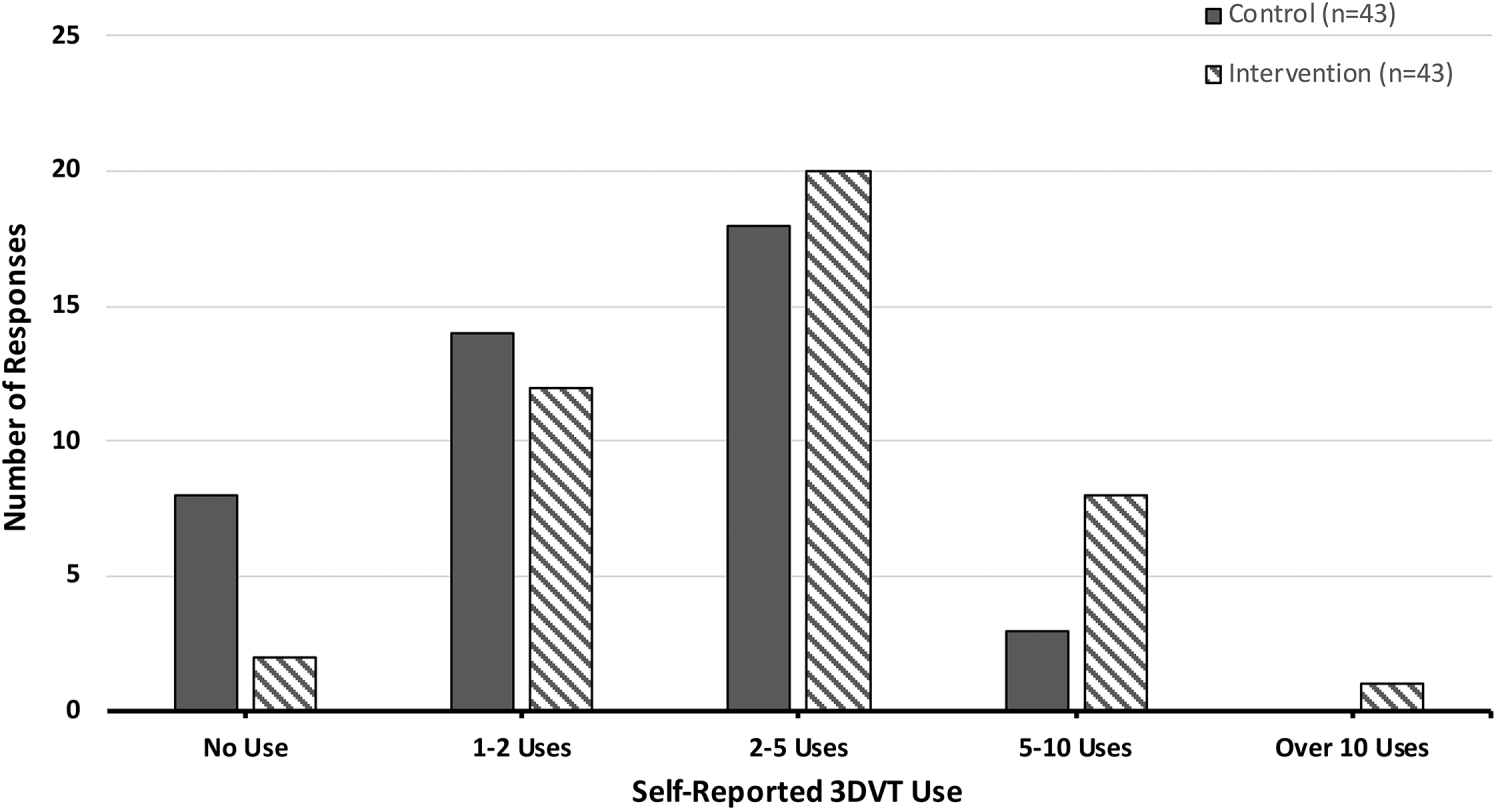
Comparison of self‐reported 3DVT use during the reproductive system unit between control group (*n* = 43, mean rank = 37.49, missing data = 3) and intervention group (*n* = 43, mean rank = 49.51, missing data = 3) participants.

Self‐reported use data was further analyzed between control and intervention groups per academic unit, and the intervention was found to be a significant factor for the reproductive system only. An independent‐samples Mann–Whitney test revealed intervention group participants reported using the 3DVT model significantly more for unit 1, the reproductive system (*Mdn* = 2, *SD* = 0.86) than the control group (*Mdn* = 1, *SD* = 0.92, *U* = 1183, *z* = 2.38, *p* = 0.017, *r = 0*.26) (Figure 4).

**FIGURE 5:**
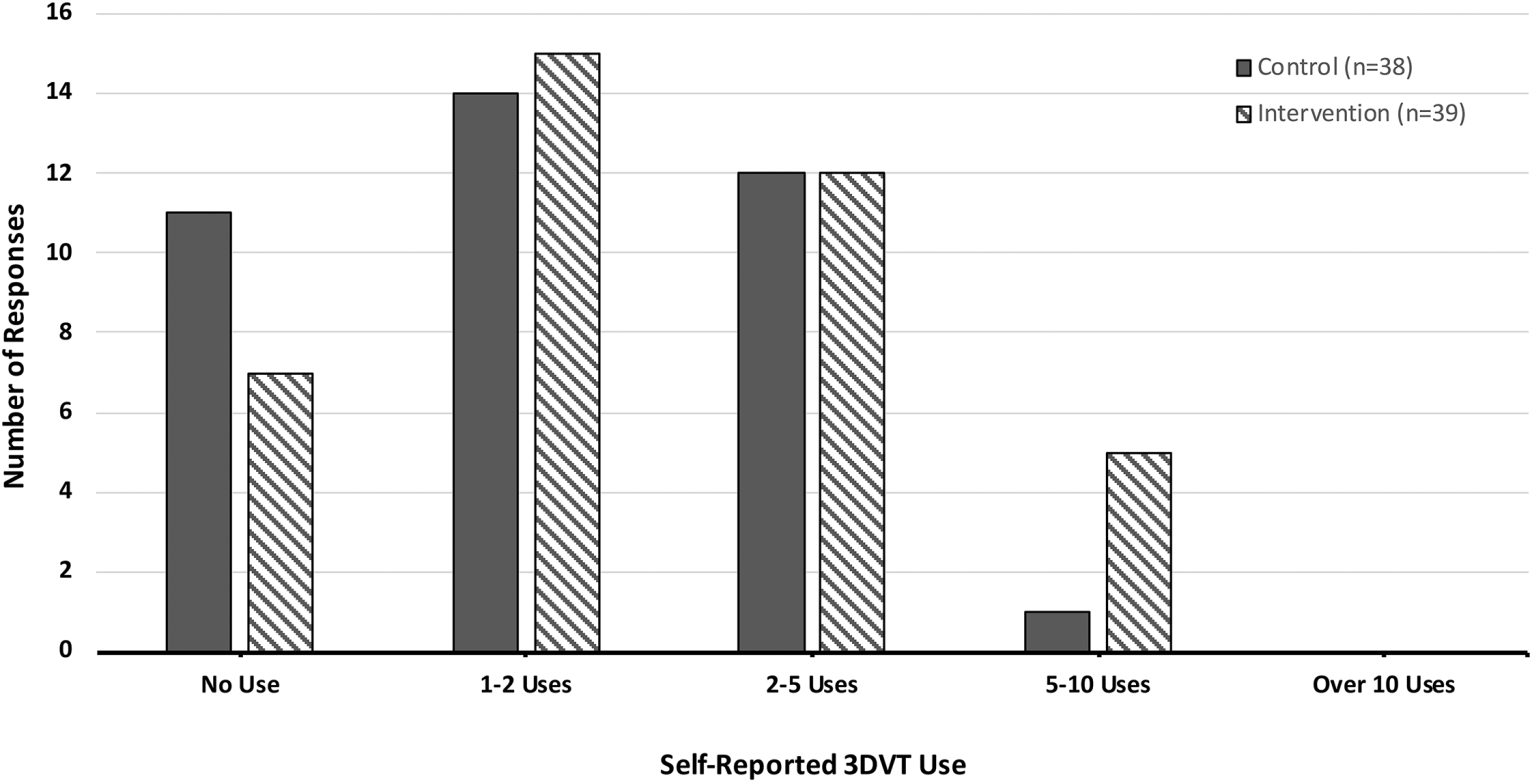
Comparison of self‐reported 3DVT use during the endocrine system unit between control group (*n* = 38, mean rank = 35.66, missing data = 8) and intervention group (*n* = 39, mean rank = 42.26, missing data = 7) participants.

This difference was not observed for the endocrine system unit. In this case, both the intervention (*Mdn* = 1, *SD* = 0.91) and control (*Mdn* = 1, *SD* = 0.85) participants reported 3DVT use that was not significantly different (*U* = 868, *z* = 1.36, *p* = 0.17, *r* = 0.16) (Figure 5).

### Student perception of 3DVT model

**FIGURE 6:**
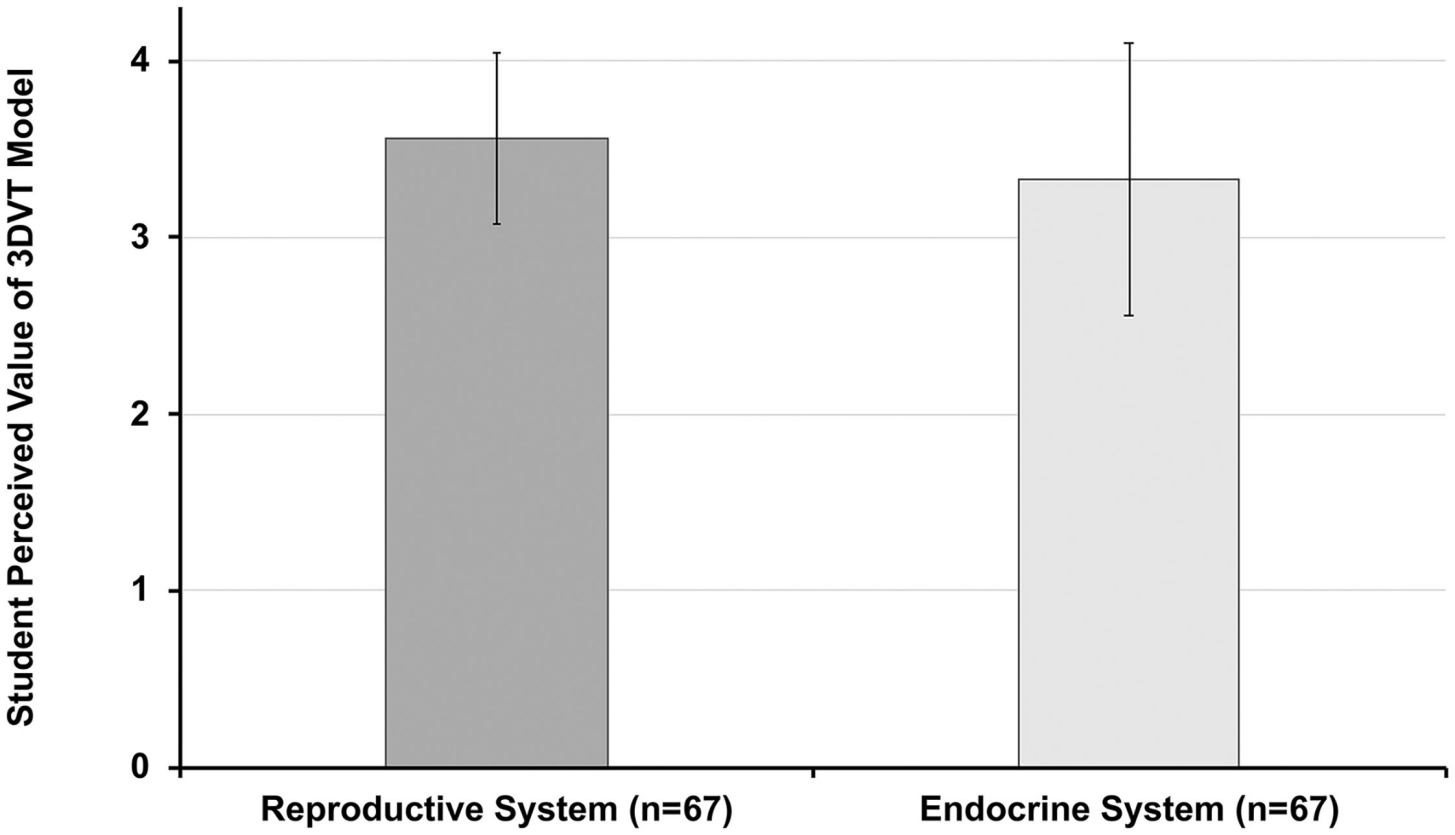
Comparison of participant assigned grades for 3DVT anatomy model value based on academic content (*n* = 67, Excluded incomplete paired data = 25). Grade scale: 4 = Mastery (A), 3 = Strong performance (B), 2 = Acceptable performance (C), 1 = Marginal performance (D), and 0 = Unacceptable performance (F). Vertical bars indicate ± standard deviation (±*SD*).

The student perceived value of the 3DVT model for the reproductive system was 3.56 (A−) on a 4.0 GPA scale (*SD* = 0.49, *n* = 67, Excluded incomplete paired data = 25). The student perceived value of the 3DVT model for the endocrine system was 3.33 (B+) on a 4.0 GPA scale (*SD* = 0.77, *n* = 67). Students perceived that the 3DVT model was significantly more valuable for learning the reproductive system than it was for learning the endocrine system (*t*(66) = 2.329, *p* = 0.023 using a Bonferroni correction (*p* < (0.05/2) = 0.025)), with a small effect size (*d* = 0.29) (Figure 6).

To gauge the participant perceived strengths and weaknesses of the 3DVT model, Survey 3 contained an open‐ended survey item asking all participants “Which parts of the 3D anatomy model were useful or beneficial to you? Which parts were not useful or problematic?” Seventy‐four responses (80% response rate) were collected resulting in a total of 141 coded narratives organized into five primary categories (Table 2).

**TABLE 2:** Frequency distribution of responses within the coding schema.

Primary code categories	Number of entries (%) (*n* = 74)	Emergent themes within category	Number of entries (% within category)
3DVT advantages	75 (53%)		
		Interactions: rotation, tools	8 (10.7%)
		Visual: 3D, ghost/hide, labels	35 (46.7%)
		Other (e.g., value and importance)	32 (42.6%)
3DVT challenges	17 (12%)		
		Access/Technical difficulties	7 (41.2%)
		Navigation	6 (35.3%)
		Complexity	4 (23.5%)
Lecture versus Lab	8 (6%)		
Academic content	24 (17%)		
3DVT intervention	17 (12%)		
Total entries	141 (100%)		

*Note*: Coding schema derived from Survey 3 participant responses (*n* = 74, 80% response rate).

Student perceived importance of all major curricular resources available in the course was examined for any differences between control and intervention group participant opinions. Comparisons made using independent‐samples Mann–Whitney test revealed that the intervention did not play a role in participant opinions about the importance of the 3DVT anatomy model, nor any other available curricular resource as summarized in Table 3.

**TABLE 3:** Control versus intervention group perceptions about curricular resources.

Curricular resource	Reproductive system				Endocrine system			
	*U*	*z*	*p*	*r*	*U*	*z*	*p*	*r*
Textbook	587	−1.08	0.28	−0.13	625.5	−0.613	0.54	−0.07
Lecture notes	706	0.385	0.70	0.04	762.5	1.20	0.23	0.14
3DVT model	663	−0.192	0.85	−0.02	709	0.322	0.75	0.04
Digital study area	661.5	−0.209	0.84	−0.02	645.5	−0.388	0.70	−0.05
Homework	639.5	−0.482	0.63	−0.06	724.5	0.528	0.60	0.06

*Note*: Independent‐samples Mann Whitney test findings comparing control (*n* = 40, Missing data = 6) and intervention (*n* = 34, Missing data = 12) group participant opinions. *U* = Mann–Whitney test statistic, *z* = standardized test statistic, *p* = probability value, and *r* = Pearson's correlation coefficient. Control *n* = 40, Intervention *n* = 34.

Additionally, student perceptions about the importance of the 3DVT model were compared to the other curricular resources using related‐samples Wilcoxon signed‐rank test. Frequency distributions of participant responses were used to interpret significant findings. These comparisons revealed that students found some other curricular resources to be significantly more important than the 3DVT anatomy model (Table 4). The only exception was that the 3DVT model was perceived as being significantly more important than the textbook for the reproductive system (*p* = 0.03). The 3DVT anatomy model was perceived as having equivalent importance to the textbook for the endocrine system (*p* = 0.83). Table 4 summarizes the results of these comparisons by academic unit.

**TABLE 4:** Comparison of 3DVT to other curricular resources across participant groups per academic unit.

Reproductive system (*n* = 74)				Endocrine system (*n* = 74)			
Resource comparison	*T*	*p*	*r*	Resource comparison	*T*	*p*	*r*
3DVT > Textbook	644.5	0.03*	0.25	3DVT = Textbook	568	0.83	−0.02
3DVT < Lecture notes	14	<0.001*	−0.78	3DVT < Lecture notes	0	<0.001*	−0.79
3DVT < Digital study area	379.5	0.01*	−0.30	3DVT < Digital study area	208.5	<0.001*	−0.47
3DVT < Homework	77	<0.001*	−0.66	3DVT < Homework	27.5	<0.001*	−0.70

*Note*: Participant *n* = 74, Missing data = 18, *T* = Wilcoxon's related‐samples signed‐rank test statistic, *p* = probability value, *r* = Pearson's correlation coefficient.

* Significant finding, alpha level 0.05.

### Relationship between the intervention and unit test grades

Prior to comparison of unit test grades, academic equivalence between the control and intervention groups was evaluated. Levene's test for equality of variances (*F*(88) = 0.441, *p* = 0.509) confirmed that the assumption of homogeneity of variances was tenable. On average, participants in both the control (*M* = 2.70, *SD* = 0.91) and intervention groups (*M* = 2.53, *SD* = 0.80) performed equally in the prerequisite course. The difference in the baseline academic performance between control and intervention groups was not significant (*t*(88) = 0.964, *p* = 0.338, *r* = 0.01); establishing that any differences in grades observed in this study were likely not attributable to different levels of academic ability demonstrated by control and intervention group participants.

Independent *t‐tests* of unit test grades were compared between groups to determine whether use of the 3DVT model as a teaching tool (i.e., the intervention) affected student grades. Levene's test confirmed the assumption of homogeneity was met for both unit 1 (*p* = 0.55) and unit 2 comparisons (*p* = 0.54). On average, participants in both the control (*M* = 64.7, *Mdn* = 61.2, *IQR* = 21.15, *SD* 13.81) and intervention group (*M* = 64.94, *Mdn* = 63.5, *IQR* = 23.6, *SD* = 14.05) performed equally on the reproductive system test, despite the intervention group seeing the 3DVT model used during class time (*t*(90) = −0.08, *p* = 0.94, *r* = 0) (Table 5). This lack of difference in academic performance between the control (*M* = 73.51, *Mdn* = 74.2, *IQR* = 22.55, *SD* = 14.55) and intervention groups (*M* = 71.95, *Mdn* = 71.7, *IQR* = 20, *SD* = 13.15) test performance held true for the endocrine system unit test (*t*(85) = 0.53, *p* = 0.60, *r* = 0).

**TABLE 5:** Comparison of unit test grades.

Unit test grade	Control			Intervention			*t*	*p*	*r*
	*n*	*M*	±*SD*	*n*	*M*	±*SD*			
Unit 1	46	64.70	±13.81	46	64.94	±14.05	*t*(90) = −0.08	0.94	0
Unit 2	44	73.51	±14.55	43	71.95	±13.15	*t*(85) = 0.53	0.60	0

*Note*: Participant test scores (%) on unit 1: Reproductive system (control *n* = 46, intervention *n* = 46) and unit 2: Endocrine system (control *n* = 44, Missing data = 2; intervention *n* = 43, Missing data = 3). Grading schema = 60% passing with marginal performance (letter grade = D) and 70% passing with adequate performance (letter grade = C). *M* = mean percentage score, ±*SD* = ±standard deviation, *t* = *t*‐test statistic, *p* = probability value, and *r* = Pearson's correlation coefficient.

### Internal check for bias in the experimental design

Pearson's chi‐square test was used to evaluate the nominal responses (i.e., yes, no, and unsure) regarding exposure to the 3DVT anatomy model in the laboratory section. Control and intervention group participants differed in their self‐reported exposure to the 3DVT model during the laboratory section of the course for both the reproductive system (*X* ^2^ (2, *N* = 85) = 14.38, *p* < 0.001) and the endocrine system (*X* ^2^ (2, *N* = 77) = 22.13, *p* < 0.001).

More intervention group members reported 3DVT use in the laboratory section than reported by the control group members (Table 6). Additionally, there was a difference in the degree of certainty regarding whether or not the 3DVT model was used in the laboratory. Following the first academic unit, more participants in the control group were uncertain about whether or not the 3DVT model was being used in laboratory (30.4% of participants), compared to the intervention group (6.5% of participants). Following the second academic unit, the degree of uncertainty in the control group remained relatively stable (28.3%), whereas the degree of uncertainty among the intervention group participants dropped to zero (0%).

**TABLE 6:** Summary of participant responses to survey item: Did your laboratory instructor use the 3DVT anatomy model in the laboratory for this unit, the (reproductive/endocrine) system?

	Unit 1: Reproductive system				Unit 2: Endocrine system			
	Control		Intervention		Control		Intervention	
	Frequency	Percent	Frequency	Percent	Frequency	Percent	Frequency	Percent
Yes	11	23.9	27	58.7	10	21.7	28	60.9
No	17	37	13	28.3	15	32.6	11	23.9
Unsure	14	30.4	3	6.5	13	28.3	0	0
Missing	4	8.7	3	6.5	8	17.4	7	15.2
Total	46	100	46	100	46	100	46	100

*Note*: Total respondents for reproductive system *n* = 85 (Missing data: control group = 4, intervention group = 3). Total respondents for endocrine system *n* = 77 (Missing data: control group = 8, intervention group = 7).

## DISCUSSION

The findings of this study represent a contribution to research regarding voluntary student access and use of 3DVT anatomy models. Participants in both the control and intervention groups used 3DVT anatomy models outside of the course (i.e., on their own time for their own reasons) as a self‐selected resource.

### Participant demographic information

Overall, the demographics of the participants were typical for a community college student population. In terms of gender identity distribution, the majority of participants identified as female. This skewed distribution is characteristic of higher education institutions.^70^ In terms of age, nationally 63% of higher education students are 18–24 years old.^70^ Fifty‐two percent of participants in this study were in that age range, with the average participant age being 25.5.

Ninety‐one percent of participants in this study had nonacademic responsibilities such as the need to work for pay, which is characteristic for US community college students.^70^ The increased nonacademic responsibilities of the participants in this study such as working for pay were not viewed as a threat since if they had any effect, they would likely reduce voluntary use of a 3DVT anatomy model rather than artificially inflate use data.

### Description of data sources describing 3DVT use

Two data sources were used to analyze participant voluntary use of the 3DVT anatomy model: Self‐reported measures and behavioral measures (i.e., login event). These measures have been found to be distinct rather than redundant measures.^71^ Findings from this study also found these data sources to be distinct. CMS‐derived data regarding the number of logins to the 3DVT model were different from the number of self‐reported uses. Modest association between self‐reported digital media use and login measures is common in research.^72^ The login events outnumbered self‐reported use approximations. Logic would suggest that the more a participant logs in to the 3DVT model, the more it is used. But increased login events could also indicate technical issues such as a bad internet connection or various types of interruptions. Additionally, not all use is equivalent. There are various degrees of use ranging from exploratory to reference to focused study. It is likely that the self‐reported use metrics in this study represented perceived meaningful use and not merely a login event. Despite the differences in the two data sources, both sources confirmed that participants (both control and intervention groups) accessed and used 3DVT voluntarily, outside of the classroom.

### Effects of the intervention

In this study, the intervention of using a 3DVT anatomy model for teaching did not consistently affect participant behavior. This contradicted the proposed hypothesis that increased familiarity with 3DVT would increase 3DVT use. This finding supports the notion that student familiarity with a digital resource alone may not be enough of an influence to increase student use.^73^ The correlation between familiarity and digital media usage has yielded inconclusive research findings. While some studies suggest that familiarity promotes digital resource use,^44^ others indicate it has no significant impact.^73^

The intervention group participants reported 3DVT model use in the laboratory over double the amount reported by control participants, despite the fact that all of the laboratory sections had a consistent teaching design and participants were randomly enrolled among the laboratory sections. Therefore, the difference in student perception between the control and intervention groups could not be attributed to an actual difference in 3DVT use in the laboratory. Nevertheless, this difference in student perception was not viewed as a threat to internal validity since intervention group participants were also exposed to the 3DVT model during lecture. One possible explanation for intervention group participants reporting more 3DVT use in the laboratory sections could be that they recognized 3DVT models better than control group participants. This conjecture is supported by the high level of uncertainty about whether or not the 3DVT model was used in the laboratory expressed by control group participants. If intervention group participants were better able to identify 3DVT use in the laboratory that could be an important advantage provided by the intervention. Generally speaking, if students are unsure about a particular curricular resource (as seen with control group participant uncertainty about the 3DVT model), then they could be missing out on a valuable learning tool.

### Academic content and participant 3DVT use

It is possible that 3DVT anatomy models are better for achieving specific learning goals as opposed to all learning goals.^11^, ^74^ This may be unsurprising given that it is generally accepted that any one resource may not be equally beneficial across all learning objectives.^75^, ^76^, ^77^ In anatomy education, a variety of curricular resources (e.g., digital and traditional) and pedagogic practices (e.g., lecture and laboratory) work best.^41^, ^78^ For example, numerous studies have shown that 3DVT use may be more effective for spatial knowledge acquisition compared to other educational methods.^21^

In this study, the nature of the academic content was a significant factor in self‐reported 3DVT use when analyzed across groups. This is understandable since there are many digital learning tools available in anatomy education with different strengths and weaknesses, so particular digital resources such as 3DVT may support some learning objectives better than others. For example, learning topics related to anatomy (e.g., physiology, surgical procedures, and clinical skills) may be better supported using different digital platforms such as simulated laboratories and simulators.^79^, ^80^, ^81^

Open‐response survey data suggested that student preference to use 3DVT was related to the nature of the academic content. For example, when asked about the value of the 3DVT model, one participant stated “Honestly, it depends on the unit we are in.” For these participants, the interconnected and localized nature of reproductive system anatomy was more aligned with the strengths of 3DVT compared to individual and diffusely located organs of the endocrine system. Even though the endocrine system is strongly related to the reproductive system (with the gonads being part of both systems), participants explained that the 3DVT model was very effective for visualizing the reproductive tract because it is a continuous tract of interconnected structures. A few participants responded that the 3DVT model was beneficial for learning the reproductive system specifically, whereas no participants specifically mentioned the endocrine system.

### Student perceived affordances of 3DVT models

Overall, participants viewed using 3DVT very positively with 53% of open‐ended responses identifying various advantages of using 3DVT. This aligns with other findings that have shown student preference for using 3DVT to learn anatomy.^25^ Advantages cited by participants included 3DVT features such as visual superiority and interactivity. Four specific visual affordances were repeatedly voiced by the participants: the three‐dimensional nature of the tool, the ability to make fine distinctions using highlights, textual information that could be toggled on and off, and the ability to make a region transparent or invisible revealing contiguous anatomical structures. Participants noted that the ability to freely rotate and zoom was a meaningful experience that was uniquely available from the 3DVT model compared to other resources at their disposal. Additionally, the 3D imagery used during class helped participants understand the connections between organs from different angles. “It's a lot easier for me to grasp a 3D model than a flat image.” Another participant talked about the 3DVT model reinforcing a mental picture, in other words, assisting the learning process. The visual aspect of the 3DVT model was described by participants as being realistic and enhancing understanding and recall.

### Student perceived challenges of 3DVT models

Of course, there were also challenges identified by participants such as increased cognitive load and technical difficulties. It was not expressed by participants that the 3DVT model hindered learning or negatively affected grades. This is notable because research findings suggest that the spatial and visual abilities of the learner play a role in the effectiveness of 3D learning tools.^22^, ^32^, ^82^ This lack of criticism could be associated with the fact that in this study, 3DVT use was completely optional, so if a student did not perceive value or had difficulty using 3DVT then they likely used a different learning method.

Participant spatial ability was not measured in this study, but using 3DVT for teaching provided guidance to students of all spatial abilities with limited anatomical knowledge about how to interpret structural relationships that were visible using a 3DVT anatomy model. Additional guidance interpreting academic content has been shown to increase student understanding.^83^, ^84^ As one participant put it, “just looking at [the 3DVT model] isn't necessarily super helpful, so you need … more depth.” This problem was echoed by participants who found the 3DVT model too complex to use on their own after class, “it was too complicated for me to understand.” Learning to use digital resources introduces additional cognitive load.^85^, ^86^, ^87^ The rotation function of the 3DVT model was very precise, so that could have played a role in a participant inability to locate anatomy of interest or find an optimal model and angle. In summary, having the educator explain the anatomy visible on the 3DVT model during class may have provided needed direction for some students who found it too complex to use without seeing the 3DVT anatomy model in action first. If participants wanted to use a 3DVT anatomy model on their own for study purposes, they had to learn how to use it, which was not a learning objective of the course.

Technical issues were also described by participants. These issues ranged from forgetting a password to forgetting how to access the 3DVT model to difficulty navigating to an area of interest. Human anatomy is a complex subject, so combined with increased nonacademic responsibilities, access issues were sometimes insurmountable, “I had difficulty accessing it, so I did not use it.” Even for those who successfully logged into the 3DVT model, glitches like long loading times and the continued need to refresh a page prevented some participants from using the 3DVT model. Also, 3DVT is not an ideal technology for use on a smart phone, and this was the type of device some participants used to access the 3DVT model. “It was a little hard to work on my phone and figure out the buttons…” Navigation proved to be a challenge and presented a struggle for some participants.

### Student perceptions: 3DVT compared to other curricular resources

Although students had on overall positive opinion about the 3DVT anatomy model, when the 3DVT model was placed in context with other available curricular resources it was largely considered less important. Most other resources utilized within the course (lecture notes, digital study area, and homework assignments) were perceived as being significantly more important than the 3DVT anatomy model. Curricular resources such as homework assignments and quizzing have been shown to be learning methods valued by students.^88^, ^89^, ^90^ The 3DVT model was considered significantly more important than the textbook for the reproductive system, but equivalent to the textbook for the endocrine system. This finding reinforces the notion that the value of a 3DVT anatomy model may depend on the nature of the academic content. It may also suggest that curricular resources customized specifically for the learning objectives of the course such as lecture notes and homework assignments may be more valuable to students compared to non‐customized resources such as a textbook or a 3DVT anatomy model.

### Intervention and grades

Previous studies have demonstrated an association between student 3DVT use and increased grades.^91^, ^92^, ^93^ This study looked for a correlation between using 3DVT for teaching and student grades. No significant difference in grades existed between the intervention and control groups for either unit test, suggesting that teaching with 3DVT may not improve student academic performance. Despite this, participants perceived learning and had overall positive impressions of the 3DVT anatomy model.

Many factors affect academic performance, such as student ability to manage their study load, application of efficient study techniques, and time management.^94^, ^95^ Since this was an in situ study, covariates that affected grades were not controlled. Therefore, although this study did not show a relationship between the intervention and grades, it remains a possibility that individual students improved their understanding and academic performance in part because a 3DVT anatomy model was being used as a teaching tool.

### Considerations for pedagogy

Spatial ability is not a fixed aptitude, it can change over time.^96^ Research findings suggest that exposure to 3D academic content can improve student spatial ability.^97^ Therefore, there is a possibility that using a 3DVT anatomy model for teaching while providing visual and verbal guidance has the potential to bridge the gap between low and high‐spatial ability learners since the educator is providing explanation throughout the changing 3D visuals.^84^, ^98^ This practical suggestion is similar to Hoyek et al.^99^ who found that 3D animation combined with a description from the educator facilitated learning complex academic content.

The Covid‐19 pandemic that caused the premature end to this study also highlighted a known advantage of monoscopic 3DVT anatomy models: availability outside the walls of the physical laboratory. Physical models are traditional and effective learning tools,^100^ although they are typically bound to a specific location such as the laboratory or library that have limits on availability. Because of this, physical models are not necessarily available at a time and place convenient for the learner. This may also be true of stereoscopic digital anatomy models since specialized equipment (e.g., VR headset, haptic devices, dual projection with polarizing filters, specialized viewing glasses) is needed. The additional equipment required to use stereoscopic anatomy models may make these tools less convivial for use outside of the classroom or laboratory, limiting them similarly to physical models and specimens. The availability of monoscopic 3DVT anatomy models outside of the laboratory provided a consistent and reliable curricular resource during and after this study, despite the institutional facility closure due to the pandemic. Inclusion of monoscopic 3DVT anatomy models within anatomy education could work to stabilize the curriculum and make it more sustainable and accessible.^101^, ^102^ This could be especially true for student populations that have increased nonacademic responsibilities requiring them to study in ways and at times most convenient for them.^103^ Integrating 3DVT into pedagogy could be done in many ways, for example, integrating 3DVT into teaching methods and recommending 3DVT as a supplemental resource.^38^

### Implications and future research

In summary, the findings of this study provide evidence that community college HAP students utilize 3DVT anatomy models as an optional resource whether they are familiarized with the resource during lecture or not. Participants had a largely positive perception of 3DVT anatomy models, although there were differences in the perceived value of 3DVT anatomy models based on the nature of the academic content. Overall, participants in the intervention group reported using 3DVT significantly more than participants in the control group for the reproductive system only, which suggests that use may depend on the nature of the academic content. Simply integrating 3DVT models as a teaching tool may not be enough to encourage student use. Using 3DVT anatomy models for teaching helped participants identify 3DVT models outside of class, as evidenced by the level of certainty intervention group members had about whether or not 3DVT was being used in the laboratory.

These findings could serve as a basis for administrative considerations regarding investment in technology, including at an institutional level. It is possible that these findings could be applicable to a broader student population. Even though this study looked at community college HAP students in nursing and allied health majors, it is feasible that 3DVT anatomy models might also be utilized by student populations in other programs such as art, health, and physical education.^104^, ^105^

It is also reasonable to consider implications of this study for digital resources other than 3DVT. If an educator values a particular digital resource, they should consider using it for teaching purposes in addition to their typical teaching tools. It may be more effective than just recommending the resource to students. Educator use of recommended digital resources during class gives students the opportunity to gauge the benefits and downsides of the resource in context of particular academic content. This practice could enable individual students to make more informed decisions when choosing their study resources which could enable more effective use of their study time. But it would also necessitate investment and commitment to training educators in effective uses of digital resources.^31^

Further research is needed to determine which anatomical characteristics or nature of the learning goals defines the most effective use of 3DVT anatomy models for teaching and learning. More research is needed to determine if the student perceived value of 3DVT anatomy models could be improved across academic units by developing guided activities. Digital activities that specifically serve learning objectives of the course may enhance student success.^106^ Other contributing factors such as alignment with the learning objectives,^107^ alignment with the assessment modality,^108^ compatibility with the nature of the academic content,^109^ and compatibility with learner characteristics^22^, ^32^ are likely considerations involved in educator and student choice of digital supplemental resources.

### Limitations

The study was intended to run the length of one semester; however, the courses were transitioned to online learning due to the Covid‐19 pandemic. The decision to conclude the study early allowed the completion of data collection regarding 3DVT use and perceived value of 3DVT for specific academic content before any dramatic change to the course modality compromised the establishment of control and intervention groups. It also avoided any potential influence that the modality change to online learning might have on student use of the 3DVT model. All findings presented in this study regarding use of 3DVT are based on data collected prior to the disruption.

Although situating the study within an authentic educational setting provided some advantages, it also presented challenges. One challenge was defining student use of the 3DVT model. It was not possible to know exactly how the 3DVT model was utilized. Therefore, any specific effect that student 3DVT use had on learning cannot be isolated. The CMS report documented that the 3DVT model was opened by the participant but it did not provide any description of use. This is one of the reasons that multiple use data sources were analyzed in this study.

The 3DVT characteristics that participants defined as meaningful were measured in this study using student responses to qualitative and quantitative inquiries, but participant behavior regarding 3DVT use was not directly observed. Self‐reported use and perception data were collected using non‐validated surveys which had the potential of introducing bias. Future research may benefit from adapting questionnaires such as Attrackiff or the User Engagement Scale.^110^ This limitation was addressed by outlining the survey contents in the Methods section. Additionally, participant behavior and perception data were collected for only two academic units: the reproductive system and endocrine system. If the nature of the academic content played a role in the amount of 3DVT use and perception of value, then this study is limited by evaluating only two body systems.

## CONCLUSION

Anatomy models are traditional and effective tools for learning anatomy. The increased availability of monoscopic anatomy models presents an opportunity within anatomy education to make models more accessible to students at their convenience. This could be of particular importance to student populations that have limited time and academic experience. Community college HAP students reported using monoscopic anatomy models voluntarily as a supplemental resource significantly more for one academic unit compared to another, suggesting that student use of 3DVT may be dependent on the nature of the academic content rather than being consistently utilized as a learning tool across the entire course. Although 3DVT model use as a teaching tool was not shown to affect student use or academic performance, it likely played a role in student ability to recognize the technology outside of the classroom. Even though 3DVT anatomy models were not perceived as the most valuable curricular resource, they were valued and utilized by students as a self‐selected study tool. Inclusion of monoscopic 3DVT anatomy models for teaching should be considered by educators because they increase the sustainability of the curriculum. Moreover, an educator's utilization of 3DVT anatomy models for teaching may assist students to select learning tools with greater efficacy.

## AUTHOR CONTRIBUTIONS

**Yvonne M. Baptiste:** Conceptualization; data curation; formal analysis; methodology; writing – original draft; writing – review and editing. **Samuel Abramovich:** Conceptualization; data curation; formal analysis; methodology; supervision; writing – review and editing.
